# Chemical profiling of coriander, garlic and their combination to highlight the synergistic effect of the profiled compounds through in vitro and in vivo bioassays

**DOI:** 10.1002/fsn3.4384

**Published:** 2025-01-01

**Authors:** Mohamed Ali Boujbiha, Hassiba Chahdoura, Borhane Eddine Cherif Ziani, Anis Ben Hsouna, Mejdi Snoussi, Manel Ben M'hadheb, Khaldoun Bachari, Boulbaba Selmi, Miroslava Kačániová, Wissem Mnif, Guido Flamini, Habib Mosbah

**Affiliations:** ^1^ Laboratory of Bioresources: Integrative Biology and Valorization (BIOLIVAL), Higher Institute of Biotechnology of Monastir University of Monastir Monastir Tunisia; ^2^ Unité de Recherche UR17ES30 “Génomique, Biotechnologie et Stratégies Antivirales”, Institut Supérieur de Biotechnologie de Monastir Université de Monastir Monastir Tunisia; ^3^ Centre de Recherche Scientifique et Technique en Analyses Physico‐Chimiques CRAPC Tipaza Algeria; ^4^ Laboratory of Biotechnology and Plant Improvement Centre of Biotechnology of Sfax Sfax Tunisia; ^5^ Department of Environmental Sciences and Nutrition, Higher Institute of Applied Sciences and Technology of Mahdia University of Monastir Monastir Tunisia; ^6^ Department of Biology University of Hail Hail Saudi Arabia; ^7^ Laboratory of Genetics, Biodiversity and Valorisation of Bioresources, High Institute of Biotechnology University of Monastir Monastir Tunisia; ^8^ Faculty of Horticulture and Landscape Engineering, Institute of Horticulture Slovak University of Agriculture Nitra Slovakia; ^9^ Department of Bioenergy, Food Technology and Microbiology, Institute of Food Technology and Nutrition University of Rzeszow Rzeszow Poland; ^10^ Department of Chemistry, College of Sciences at Bisha University of Bisha Bisha Saudi Arabia; ^11^ Diparitmento di Farmacia Pisa Italy; ^12^ Interdepartmental Research Centre “Nutraceuticals and Food for Health” University of Pisa Pisa Italy

**Keywords:** analgesic/gastroprotective potential, antimicrobial activity, antioxidant capacity, garlic/coriander combination, HS‐SPME/GC–MS, LC‐Q‐TOF/MS–MS

## Abstract

Hydroethanolic extracts of coriander seeds (CE), garlic bulbs (GE), and their combination (CGE), were chemically profiled using HS‐SPME/GC–MS and LC‐Q‐TOF/MS–MS to assess volatile composition and to study phenolic molecules, respectively. Biological assays were conducted through in vitro and in vivo experiments to measure the EC_50_ of the antioxidant assays and the MIC/MBC/MFC values of the antibacterial/antifungal activities. Antioxidant combination Index (CI) and fractional inhibitory concentration index (FICI) values were further recorded. The acute oral toxicity, analgesic, and gastroprotective activities were evaluated in vivo on *Wistar* rats and *Swiss albino* mice. Caffeoyl quinic acid, feruloyl‐quinic acid, and caffeic acid derivatives (quercetin, apigenin, and luteolin‐*O*‐glycoside) together with monoterpene linalool, α‐pinene, and γ‐terpinene were found to be highly present in CE. Organosulfur compounds (allicin, *S*‐allyl‐l‐cysteine, allin, γ‐Glutamyl‐*S*‐allylcysteine, and allyl sulfide) were predominant in GE. All the profiled compounds were co‐present in CGE. In vivo assays responded in a dose‐dependent showing better activity mainly at 200 mg kg^−1^. Chromatographic analysis profiled various phenolic acids, flavonols and derivatives, monoterpene, and organosulfur compounds in the assessed extracts and their combinations. Bioassays' responses were found to be dose‐dependent with better scores recorded with CGE. Thus, a synergetic effect was significantly highlighted.

## Graphical abstract

The outcome of the current research suggested that the combination of CE and GE indicated as CGE can exhibit some synergistic effect observed when tested in vitro (antioxidant and antimicrobial activities) and in vivo (analgesic and gastroprotective activities).

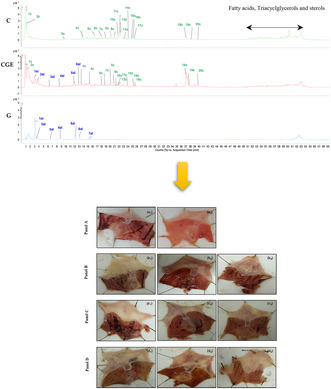

## 1 INTRODUCTION

Many cooking recipes are based on spice combinations and these mixtures are used to improve nutritional and organoleptic requirements. Furthermore, spice combination due to their content in bioactive compounds can promote health, boost health welfare, and prevent or mitigate the risk of promoting diseases (De Araújo et al., 2019; Katarzyna & Katarzyna, 2016; Opara & Chohan, 2014). Recently, there has been a substantial rise in the number of laboratory‐based experiments to identify spice bioactive compounds in order to understand the mechanism(s) behind their claims in food (Graf et al., 2005; Shahidi & Ambigaipalan, 2015). Besides, food manufacturers became increasingly aware of the interest of certain spice combinations as promoting matrices to develop novel functional foods (Katarzyna & Katarzyna, 2016). This concept is strengthening by the fact that some daily used spices contain bioactive compounds that can possess pharmacological properties (Asfaw, 2019; Guiné & Gonçalves, 2016) which was concretely reflected in the Mediterranean diet that is based on a variety of spicy foods (Renna et al., 2015). The considerable health benefits of certain spicy Mediterranean food may be in part related to the use of coriander *Coriandrum sativum* L. and garlic *Allium sativum* L. that are frequently found together in one dish. Therefore, at molecular level, the synergistic properties of those spices mixtures through their compounds interactions were previously highlighted (Bag & Chattopadhyay, 2015; Purkait et al., 2020). These interactions may be a crucial study track to explore their benefits through a deep research validation (Wang et al., 2011, 2013). Besides their roles as flavoring agents and food preservatives, garlic and coriander have the ability to reduce microbial development, control inflammation, and possess antidiabetic, analgesic, anti‐ulcer, and anticancer properties (Prachayasittikul et al., 2018; Shang et al., 2019; Wei et al., 2019). Previous research has explored the molecular mechanisms behind the individual biological activities of garlic and coriander as kitchen spices. Many authors related their biological potentials to their phenolic compounds (Batiha et al., 2020; Wei et al., 2019) that are structurally assorted leading to a variety of molecular interactions. Garlic bulbs were previously reported to contain organic sulfides (*S*‐allyl‐cysteine, diallyltrisulfide, diallyl disulfide, diallyl sulfide, allicin, alliin, ajoene, and saponins), phenolic compounds, and polysaccharides which are accountable of their health benefits sought (Batiha et al., 2020; Shang et al., 2019). The flavoring and preservative proprieties of the organic sulfides allow garlic to be a popular ingredient widely used in spicy foods. Coriander is also an important spice, mainly cultivated for the seeds and the leaves and commonly used in many preparations (Prachayasittikul et al., 2018). Scientific works (Shahwar et al., 2012; Wei et al., 2019) reported various bioactive compounds in coriander such as flavonoids, essential oils, monoterpenes, limonene, γ‐terpinene, *p*‐cymene, α‐pinene, citronellol, borneol, geraniol, camphor, dihydrocoriandrin, coriandrin, and coriandrons A‐E.

So far as one knows there are no further studies about the garlic and coriander combination as a one‐spice matrix. For this purpose, the current work attempts to study the spices extracts assessed individually and to determine the interaction effect of those spices being mixed in pair. Thus, the phytochemical profiling of volatile and phenolic molecules was performed by HS‐SPME/GC–MS and LC‐Q‐TOF/MS–MS to provide deeper insights into sample composition in order to correlate with the biological responses assessed afterward in vitro on the antioxidant and antimicrobial potential, then in vivo on acute toxicity, antiulcer, and the antinociceptive activities using animal experimental models.

## 2 MATERIALS AND METHODS

### 2.1 Chemicals

Folin–Ciocalteu's phenol reagent (FC reagent), iron (II) chloride (FeCl_2_), iron (III) chloride (FeCl_3_), potassium ferricyanide [K_3_Fe(CN)_6_], sodium chloride (NaCl), aluminum chloride (AlCl_3_), sulfuric acid (H_2_SO_4_), formic acid (HCOOH), acetic acid (CH3COOH), hydrochloric acid (HCl), sodium hydroxide (NaOH), acetonitrile (HPLC grade), ethanol (HPLC grade), 2,2‐diphenyl‐1‐picrylhydrazyl (DPPH), *β*‐carotene, linoleic acid, trichloroacetic acid (TCA), butylated hydroxytoluene (BHT), lysine acetylsalicylic acid (ASL), gallic acid (98%), rutin, and catechin were purchased from Sigma‐Aldrich.

### 2.2 Plant material and extract preparation

Coriander seeds and garlic bulbs were obtained from the local market (M'Saken‐Tunisia). These spices were authenticated by taxonomists from the Higher Institute of Biotechnology of Monastir (ISBM), Tunisia. Voucher specimens were recorded in the Herbarium of the Laboratory of Bioresources: Integrative Biology and Valorization, ISBM‐Monastir, Tunisia, under numbers Cor20 and Gar20 for coriander seeds and garlic bulbs, respectively. After shade drying, the samples were powdered in a Willy Mill to 60‐mesh size and subjected separately and in combination (1:1 w/w) for solvent extraction (80% ethanol for 24 h). The obtained extracts were filtered and evaporated at 45°C using rotary evaporator. The residual extract was freeze‐dried until obtaining a dry extract. The extracts of *C*. *sativum* seeds (CE), *A*. *sativum* bulbs (GA), and their combination (CGE) were suspended in ethanol/water (80/20, v/v) to form a stock solution of 200 mg mL^−1^ used for subsequent analyses.

### 2.3 Phytochemical analysis

#### 2.3.1 Identification of volatile compounds

The volatile fraction of the extract was analyzed using a GC–MS equipment after a headspace solid‐phase microextraction (HS‐SPME) using a Supelco SPME device (polydimethylsiloxane, PDMS, 100 μm). Sampling was achieved using the same fiber for all the analyses. After equilibration period, the headspace was sampled for 15 min and then the fiber was inserted into the GC–MS injector. Chromatographic analyses were carried out using a Varian CP‐3800 gas‐chromatograph equipped with a DB‐5 capillary column (30 m × 0.25 mm; coating thickness 0.25 μm) and a Varian Saturn 2000 ion trap mass detector. The temperature of injector and transfer line was set at 220°C and 240°C, respectively, while the oven temperature was arranged from 60°C to 240°C at 3°C/min. Helium was used as carrier gas (1 mL/min); splitless injection. Identification of the volatiles compounds was carried when comparing their retention times, their linear retention indices relative to the *n*‐hydrocarbons series, and their mass spectra with those of pure standards components and MS literature data.

#### 2.3.2 LC‐Q‐TOF/MS analysis

Phenolic profiling of the extracts was performed on a 1290 chromatograph (Agilent Technologies) coupled with Q‐TOF mass spectrometer 4600® equipped with electrospray ionization, ESI source (DuoSpray). The column was a RFC18 (20 cm, 2.1 × 100 mm i.d., 1.8 μm) set at 25°C and used for the molecular separation. The mobile phase consisted of acidified ultrapure water (0.1% formic acid, pH = 3) as phase A and HPLC grade acetonitrile as phase B, proceeding with at a flow rate of 0.5 mL/min under gradient condition: 0–5 min (5% B); 5–15 min (5–30% B); 15–30 min (30–50% B); 15–25 min (50–95% B); 30–60 min (95–95% B). The injection volume was 10 μL. The mass spectrometer (MS) was set in full‐scan m/z 100–1500 and information‐dependent acquisition (IDA) MS/MS modes, with both positive and negative ion modes. The collision energy was −70 eV. The temperature and ion spray voltage floating were 500°C and 5000/−4500 V, respectively. Mass Hunter 4.0 software was used for qualitative analysis and data acquisition. Pics tentative identification was set basing on the MS/MS fragmentation pattern and PDA spectra of the eluted components validating the results with the literature findings.

#### 2.3.3 Total phenolic, flavonoids, and flavonols

The total phenolic content (TPC), the total flavonoid content (TFC), and the flavonols (TF) of the extracts were spectrophotometrically evaluated following Mekni et al. (2013), Wolfe et al. (2003), and Zhishen et al. (1999) procedures, respectively.

### 2.4 In vitro bioassays

#### 2.4.1 Antioxidant activity

To evaluate the antioxidant activity, three in vitro complementary assays were achieved to determine the EC_50_ (mg mL^−1^) values stated as the sample concentration providing 50% of antioxidant activity (Chahdoura et al., 2014). The DPPH radical‐scavenging activity was measured at 515 nm and calculated as a percentage of DPPH discoloration. Reducing power was evaluated on the capacity of the extracts concentrations to reduce ferric ions Fe^+3^ to Fe^+2^, the recorded absorbance was measured at 700 nm. *β*‐carotene bleaching inhibition assay was monitored through the linoleate free radical's neutralization that inhibits the *β*‐carotene bleaching; absorbance was recorded at 470 nm. For all assays, the EC_50_was measured by excel software after equations transformations. BHT was used as a positive control with all the assays.

An Antioxidant Combination Index (CI) was further measured to evaluate whether the antioxidant activity of the combination is synergistic, additive, or antagonistic. The stated formula *CI =* [(*D*)_*1*_/(*Dx*)_*1*_] + [(*D*)_*2*_/(*Dx*)_*2*_] was used to measure the CI as described by Ting‐Chao et al. (1994), where (D)_1_ and (D)_2_ are the EC_50_ values of two active extracts in combination; (Dx)_1_ and (Dx)_2_ are the individual EC_50_ values of each extract individually. The antioxidant interactions were identified as synergistic if CI < 1, additive if CI = 1, or antagonistic if CI > 1.

#### 2.4.2 Antimicrobial activity

The antimicrobial tests were achieved using Gram‐positive bacteria: *Bacillus cereus* (ATCC 11778), *Staphylococcus aureus* (ATCC 25923), *Enterococcus epidermidis* (CECT 231), and *Listeria monocytogenes* (CECT933) and Gram‐negative bacteria: *Salmonella enterica* subsp. *Enterica* (CECT 443), *Shigella flexneri* (CECT 4804), *Pseudomonas aeruginosa* (PAO1), and *Escherichia coli* (ATCC 35218). Furthermore, the antifungal activity was performed using the following yeast strains: *Candida tropicalis* (06–85), *Candida albicans* (ATCC 2019), *Candida krusei* (ATCC 6258), and *Candida parapsilosis* (ATCC 20019). CECT strains were provided by the Department of Microbiology and Ecology, School of Pharmacy, University of Valencia, 46100 Burjasot, Valencia, Spain. The minimal inhibition concentrations (MICs) and the minimal bactericidal/fungicidal concentrations (MBCs/MFCs) were achieved according to Snoussi et al. (2015). The MIC was reported as the lowest sample concentration to inhibit the microorganism's growth, and the MBC/MFC values were recorded as the lowest sample concentration, which resulted into a clear fluid showing no visible growth. The microbial suspensions adjusted spectrophotometrically to 10^7^ CFU/mL were used to prepare serial two‐fold dilutions in nutrient broth at concentrations ranging from 0.2 to 200 mg mL^−1^. Subsequently, nutrient broth (95 μL) was added in each well of 96 microplates and stock solutions (100 μL) of each extract. Finally, each bacterial/fungal suspension (5 μL) was added to all wells. In each plate, the first well which contains only nutrient broth (195 μL) and inoculum (5 μL) was considered as a negative control. At the end, the microplates were incubated for 24 h at 37°C. All tests were assessed in triplicate.

Once the MICs were recorded, the Fractional Inhibitory Concentration Index (FICI) was measured as previously reported by Rakholiya et al. (2015) to interpret the results following the formula: FICI = (MIC_CGE_/MIC_GE_) + (MIC_CGE_/MIC_CE_), where: MIC_CGE_, MIC_GE_, MIC_CE_ are the MIC values of the combination, garlic, and coriander extracts, respectively. Accordingly, FICI values were interpreted as synergistic if FICI ≤ 0.5, additive if FICI0.5 < Index ≤ 4, and antagonistic if FICI > 4.

### 2.5 In vivo bioassays

Animal experimentations were performed using *Wistar* rats and *Swiss albino* mice given a standard diet and water ad libitum. All procedures were approved and performed according to the European Union regulations (Directive 86/609/CEE) for animal experiments. The Ethical approval was also obtained from the Research Ethics Committee for Life and Health Sciences at the Higher Institute of Biotechnology of Monastir, Tunisia, under the reference CER‐SVS 007/2020 ISBM.

The animals were kept under an alternative cycle of 12 h light (L)/12 h dark (D) at 22 ± 2°C. Prior to the experiment, the animals were sustained overnight and kept after being prevented from eating and only to have free water access.

#### 2.5.1 Acute oral toxicity assay

The acute oral toxicity test was achieved using *Wistar* rats (80–100 g), fasted for 12 h before the assays. The extracts were orally administered on nine groups (*n* = 6) in a single dose of 100, 200, and 500 mg kg^−1^. The control group was orally given distilled water (10 mL kg^−1^). Afterward, the animals were sustained and kept under control in order to detect any toxic effect during the first 4 h. After that, mortality and side health signs (skin eruption, diarrhea, salivation, sleep, and coma) were pointed out for 72 h and later for 14 days as previously described by Chung (2006). Following these clinical observations, the LD_50_ values were deduced.

#### 2.5.2 Analgesic activity

To assess the extract's peripheral analgesic activity, formic acid‐induced writhing method was carried out as reported previously by Ishola et al. (2012). Sixty‐six *Swiss albino* mice weighing 18–25 g were divided into 11 groups (*n* = 6). Before 15 min, the negative and the positive control groups were intraperitoneally administered with distilled water (10 mL kg^−1^) and lysine acetylsalicylic acid (ASL, 200 mg kg^−1^, as reference drug), respectively. Whereas, the rats of experimental groups were injected with the three extracts at doses of 50, 100, and 200 mg kg^−1^. Subsequently, the intraperitoneal injection of the acetic acid (10 mL kg^−1^) in mice was performed to stimulate the writhing reflex. At the end, the writing's number was enumerated after 20 min.

#### 2.5.3 Gastroprotective activity

The gastroprotective activity of the extracts was studied following the method previously reported by Baiubon et al. (2016) after inducing gastric ulcer using HCl/EtOH. Sixty‐six *Wistar* rats, divided into 11 groups (*n* = 6) were fasted for 24 h prior to the test. After that, the negative and the positive control groups were injected intraperitoneally with 2.5 mL kg^−1^ of NaCl (9‰) and 30 mg kg^−1^ of omeprazole (as reference drug), respectively. Whereas, the experimental groups were received extracts at doses of 50, 100, and 200 mg kg^−1^. Thirty minutes later, a mixture of HCl/ethanol (40:60, v/v) was orally administered to all groups to induce gastric ulcer. After 1 h, the animals were sacrificed, so we could remove their stomachs quickly, open, wash, and stretch them on cork plates which facilitate to take photographs so as to evaluate the gastric lesion. The stretch of the visible stomach lesions was recorded in order to calculate the ulcer index.

### 2.6 Statistical analysis

The results are shown as the mean ± S.E.M. One‐way ANOVA and multi‐range post hoc Dunnett's test were run for data analysis. *p*‐values less than .05 (*p* < .05) were interpreted as statistically significant.

## 3 RESULTS AND DISCUSSION

### 3.1 Volatile compounds analysis

Volatile compounds are mainly responsible for the distinguished flavor of foods and are among the main parameters, which shape the quality and influence consumer attitudes (Ayseli & Ipek Ayseli, 2016). Herein, the volatile compounds of garlic bulbs, coriander seeds, and their combination were determined by HS‐SPME/GC–MS. The percentages and the retention indices of the identified components are shown in Table 1. The main chemical class in coriander seeds was the oxygenated monoterpenes (60.2%) and the monoterpene hydrocarbons (38.9%). While the sulfur derivatives (98.5%) were the principal chemical classes in garlic bulbs. These chemical classes were simultaneously recorded in combination with percentages of 50.8%, 29.2%, and 17.0%, respectively for the oxygenated monoterpenes, monoterpene hydrocarbons, and sulfur derivatives. Among the 20 detected compounds in garlic bulbs, diallyl disulfide (79.4%), methyl allyl disulfide (9.6%), allylmercaptane (4.2%), and (E)‐1‐propenyl allyl disulfide (4.0%) were the major components. Abe et al. (2020) recently reported the almost similar composition in fresh and processed garlic being majored by sulfur‐containing volatile compounds. In addition, the volatile compounds identified in coriander seeds were linalool (52.2%), α‐pinene (14.5%), γ‐terpinene (12.0%), camphor (4.5%), and limonene (3.1%); these compounds were also previously reported by Shahwar et al. (2012) with some quantitative differences. According to these authors, the variations with the current finding may be linked to the environmental circumstances, agricultural practices, and the harvest period (Shahwar et al., 2012). It is noticeable that some volatile compounds not detected in garlic bulbs and seeds coriander alone were newly appeared in the combination. The newly detected volatile compounds in the combination have been identified as 2‐vinyl‐4H‐1,3‐dithiine (1.2%), methyl (E)‐1‐propenyl disulfide (1.1%), 3‐vinyl‐1,2‐dithiacyclohex‐5‐ene (0.8%), and hexanal (0.2%). These compounds have been generated during the extraction process due to the presence of the two different spice materials in the mixture. Three of these compounds are sulfur‐containing compounds, two are cyclic and can be obtained by dimerization of allylmercaptan while the methyl (E)‐1‐propenyl disulfide can be originated from a degradation of the latter or a modification of allylmercaptan. Hexanal is considered a product of the oxidation of fatty acids; this may occur during the extraction procedure.

**TABLE 1:** Volatile compounds of garlic, coriander, and their combination.

N°	Constituents	LRI^a^	(%)^b^		
			*Coriandrum sativum*	*Allium sativum*	Combination
1	Allylmercaptane	595	nd	4.2	nd
2	Methyl allyl sulfide	697	nd	0.1	nd
3	Hexanal	802	nd	nd	0.2^c^
4	Diallyl sulfide	866	nd	0.7	0.1
5	Methyl allyl disulfide	919	nd	9.6	1.2
6	Tricyclene	928	0.1	nd	nd
7	α‐Thujene	933	0.2	nd	0.2
8	α‐Pinene	941	14.5	nd	9.9
9	Methyl (E)‐1‐propenyl disulfide	942	nd	nd	1.1^c^
10	Methyl (Z)‐1‐propenyl disulfide	948	nd	0.2	nd
11	Camphene	955	1.9	nd	1.3
12	Sabinene	977	0.8	nd	0.8
13	β‐Pinene	982	1.5	nd	1.1
14	Myrcene	993	1.3	nd	0.5
15	α‐Terpinene	1020	0.1	nd	nd
16	*p*‐Cymene	1028	2.8	nd	4.1
17	Limonene	1032	3.1	0.1	2.5
18	(E)‐β‐ocimene	1052	0.1	nd	nd
19	γ‐Terpinene	1063	12.0	nd	8.3
20	*Cis‐*sabinene hydrate	1070	0.1	nd	nd
21	*Cis‐*linalool oxide (furanoid)	1076	0.2	nd	0.3
22	Diallyl disulfide	1082	nd	79.4	3.9
23	Terpinolene	1090	0.5	nd	0.5
24	Linalool	1101	52.2	nd	45.2
25	(E)‐1‐propenyl allyl disulfide	1101	nd	4.0	nd
26	Methyl allyl trisulfide	1141	nd	0.1	2.8
27	Camphor	1145	4.5	nd	3.7
28	Borneol	1168	0.4	nd	0.3
29	4‐Terpineol	1179	0.3	nd	0.3
30	3‐Vinyl‐1,2‐dithiacyclohex‐5‐ene	1191	nd	nd	0.8^c^
31	α‐Terpineol	1191	0.2	nd	0.2
32	*n*‐Dodecane	1200	0.1	nd	0.1
33	Decanal	1206	0.1	nd	0.2
34	2‐Vinyl‐4H‐1,3‐dithiine	1207	nd	nd	1.2^c^
35	Cumin aldehyde	1241	0.1	nd	0.1
36	Carvone	1244	0.2	nd	0.2
37	Geranial	1271	nd	nd	0.1^c^
38	(*E*)‐anethole	1284	0.3	nd	0.2
39	diallyltrisulfide	1298	nd	0.2	5.9
40	*n*‐Tridecane	1300	0.1	nd	nd
41	Myrtenyl acetate	1327	0.1	nd	nd
42	α‐Copaene	1377	nd	0.1	nd
43	Geranyl acetate	1383	1.9	nd	0.4
44	β‐Caryophyllene	1419	0.2	0.2	0.3
45	Aromadendrene	1440	nd	0.1	nd
46	α‐Humulene	1455	nd	0.1	nd
47	Alloaromadendrene	1462	nd	0.1	nd
48	*Trans*‐calamenene	1527	nd	0.1	nd
49	α‐Cadinene	1537	nd	0.1	nd
	Monoterpene hydrocarbons	–	38.9	0.1	29.2
	Oxygenated monoterpenes	–	60.2	0.0	50.8
	Sesquiterpene hydrocarbons	–	0.2	0.8	0.3
	Phenylpropanoids	–	0.3	0.0	0.2
	Sulfur derivatives	–	0.0	98.5	17.0
	Non‐terpene derivatives	–	0.3	0.0	0.5
	Total identified		99.9	99.4	98.0

Abbreviation: nd, not detected.

^a^ LRI, linear retention indices (HP‐5 column).

^b^ (%): The relative proportions of the constituents obtained by peak area normalization; Major compounds in bold.

^**c**^ The new appeared compounds.

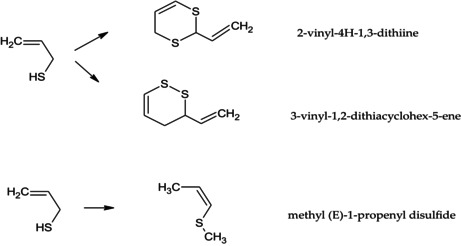

### 3.2 LC‐Q‐TOF/MS–MS characterization

**FIGURE 1:**
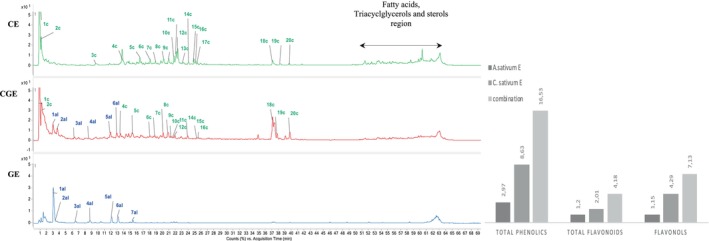
LC‐Q‐TOF profile of *Coriandrum sativum* L extract (CE), *Allium sativum* L. (GE) extract, and the combined extract (CGE). TF, total flavonols (mg Req/g extract); TFC, total flavonoid compounds (mg Ceq/g extract); TPC, total phenolic compounds (mg GAeq/g extract). Mean ± SD, *n* = 3. mg GAeq/g extract: Mg of Gallic Acid equivalents (GAeq) per g of extract. mg Ceq/g extract: Mg of catechin equivalents (Ceq) per g of extract. mg Req/g extract: Mg of rutin equivalents (Req) per g of extract.

Several phenolic compounds of the studied extracts were tentatively identified by LC‐DAD‐Q‐TOF and ion trap electrospray mass spectrometry ESI‐MS. The retention time (Rt), spectral data of molecular ion (*m/z*), main fragment ions in MS2, and compound names are outlined in Figure 1 and Tables 2, 3, 4. The two spice extracts presented different molecular profiles and diverse chemical compounds that were co‐present in the combination extract. For *C*. *sativum* extract, 15 compounds were identified in the hydroethanolic extract, where the flavones, flavanones, phenolic acids, glycoside flavonoid conjugates, and their derivatives were mainly present (Table 2). However, the majorities of the specified compounds were also detected in the combined sample formulation and were identified with the same annotation such as represented in Table 4. Peaks 1c in *C*. *sativum* extract and combination at retention time (Rt ~ 0.9 min) revealed a molecular ion [M − H]^−^ at *m/z* 341 releasing MS2 fragments at *m/z* 179 due to cleavage of hexoside moiety (162 Da) and m/z 161 [(M − H)−hexose−H_2_O]^−^, *m/z* 153, and 143 that can suggest the structure of caffeoyl‐*O*‐hexoside (Ben Said et al., 2017). Peak2c in sample CE and CGE at retention time (Rt) ~ 0.9 min produced a deprotonated molecular ion [M − H]^−^ at *m/z* 369 and fragment ions at *m/z* 195, 191, and 133 corresponding to quinic acid that can be associated with trihydroxycinnamoyl residue based on a previous report (Ruan et al., 2019), and thus, the compound was tentatively identified as trihydroxycinnamoyl quinic acid. The peak 3c at Rt ~ 9.67 min was identified as ethyl caffeate based on its deprotonated molecular ion [M − H]^−^ at *m/z* 207 and MS2 spectrum, which showed a fragment at *m/z* 161 [(M − H)−CH_3_−CH_2_OH]^−^ (Barth et al., 2019). Peaks 4c and 5c in *C*. *sativum* extract/combination presented deprotonated [M − H]^−^at *m/z* 353 and fragments at m/z 191, 179, 173, 135, and 178. The pattern of fragmentation along with the characteristic [M − H]^−^ ion at *m/z* 353 led to the identification of the compound as mono‐caffeoylquinic acid; on the basis of the fragments, we can tentatively assign the two derivatives at the 3 and 5‐*O*‐caffeoyl quinic acid, respectively (Clifford et al., 2003). Peak 6c in *C*. *sativum* extract/combination presented [M − H]^−^ ion at *m/z* 367, and MS2 fragments at *m/z* 193 [ferulic acid–H]^−^, 191 [quinic acid–H]^−^, and 134 [(ferulic acid–H)–CO_2_–CH_3_] and was tentatively identified as 4‐*O*‐feruloyl‐quinic acid (Masike et al., 2017). Peak 7c was characterized by [M − H]^−^ ion at *m/* 515 and fragment ion at 353 [(M − H)^−^ 162]^−^ corresponding to the loss of a caffeoyl moiety, and other fragments (m/z at 191, 179, 173, and 135) characteristic of the cleavage of 3,5‐*O*‐dicaffeoylquinic acid. Peak 8c presented [M − H]^−^ ion at *m/z* 499, with MS2 fragment at *m/z* 377 [(M–H) − 122]^−^, 273 [(M − H) − 226]^−^, 163 [(M − H) − 336]^−^ due to the loss of a dehydrated caffeoyl‐quinic acid unit, 119 [(M − H) − 336–44]^−^ corresponding to the loss of a coumaroyl radical, which suggest that this compound could be a caffeoyl‐coumaroyl‐quinic acid (Benayad et al., 2014). Peaks 9–11c (Rt from 20.94–22.10 min) in *C*. *sativum* extract/combination are isomers and presented pseudomolecular ions [M − H]^−^ at *m/z* 609, all of them releasing a unique MS2 fragment at *m/z* 301 (quercetin). The loss of 308 amu was coherent with a disaccharide formed by a deoxyhexoside and hexoside rutinoside moiety, and the compound may be specified as quercetin‐3‐*O*‐rutinoside and its isomers. Peak 12c presented [M − H]^−^ ion at *m/z* 477 and an MS2 fragment at *m/z* 315 [(M − H) − 162]^−^ mass units corresponding to a hexose moiety and fragment ions at *m/z* 179 and 151 produced by rings A and B of the flavone structure; the compound was tentatively identified as isorhamnetin‐*O*‐hexoside (Brito et al., 2014). Peaks 13 and 14c presented [M − H]^−^ ion at *m/z* 593 that displayed the losses of 90 and 120 u, m/z 503 [(M – H) − 90]^−^ and m/z 473 [(M − H) − 120]^−^, respectively, being identified as two isomeric forms of 6,8‐*C*‐dihexosylapigenin (Parejo et al., 2004). Peaks 15 and 16c were identified as apigenin‐*O*‐glycosylated compounds basing on the fragmentation pattern [M − H]^−^ at *m/z* 473 and MS2 at *m/z* 429 (*M*‐acetyl moiety) and the attributed name was apigenin‐acetyl‐hexoside (Brito et al., 2014). Peak 17c (Rt 25.39 min), revealed a pseudomolecular ion [M − H]^−^ at ion at m/z 609 and MS2 ions at m/z 489 [(M − H) − 120], m/z 369 [(M − H) − 240] indicating the presence of di‐*C*‐glucoside and were estimated as luteolin 6,8‐di‐*C*‐glucoside (Brito et al., 2014). MS profiling of peak 18c eluting at Rt37,03 min produced a deprotonated molecular ion at *m/z* 329 and a single fragment ion at 285 [(M – H) – 162] in the MS2 experiment related to the loss of carbon dioxide molecule. The compound was tentatively identified as carnosol (Hossain et al., 2010). Peak 19c (Rt 38.25 min) showed a pseudomolecular ion [M − H]^−^ at m/z 431 that yielded two fragment ions at *m/z* 311 [(M − H) − 120]^−^ that is a typical fragment ion of mono‐*C*‐glycosides and *m/z* 283 [(M − H) – 162 – 162]^−^, related to the loss of hexoside moieties with no loss of water molecule which is characteristic for *C*‐6‐glycoside, while the ion at *m/z* 311 was coherent with the flavonol apigenin, which allowed assigning it as vitexin (apigenin‐8‐*C*‐glucoside) (Brito et al., 2014). Peak 20c presents a molecular ion at *m/z* 925 and MS2 main fragment at *m/z* 605, corresponding to the loss an *O*‐(acyl)‐deoxyhexosyl[(M − H) − (acyl−18) − 146]^−^. Other fragment signals were observed at *m/z*563 [(M − H) − 320 − (acyl−18)]^−^, *m/z* 443 [(M − H) − 320 − (acyl−18) − 120]^−^ indicating a *C*‐glycosylation linkage with a hexose. Whereas the ions observed at*m/z* 383 and *m/z*353 corresponds to the loss of the hexosyl and a pentosyl residue. The compound can be tentatively identified as a complex flavonoid formed by luteolin, and O and C glycosidation. Checking the MS results with the literature a similar compound matching the MS data was reported in the paper of Benayad et al. (2014) as luteolin 7‐*O*‐(6″‐quinoyl)‐rhamnosyl‐6‐*C*‐pentosyl‐8‐*C*,*O*‐(6″‐acetyl)‐glucoside; due to the large number of groups and moieties, our identification cannot be ascribed to this specific compound and further study should be performed to isolate and elucidate the chemical structure of Peak20c.

**TABLE 2:** Retention time (Rt), wavelengths of maximum absorption in visible region (λ_max_), mass spectral data, and tentative identification of phenolic compounds in *Coriandrum sativum* L.

Peak	Rt (min)	[M − H]^−^ (m/z)	MS^2^ (m/z)	Tentative identification	References
**1** ^**c**^	0.91	341	179, 161, 153, 143	Caffeoyl‐*O*‐hexoside	Mekni et al. (2013)
**2** ^**c**^	1.23	369	195, 191, 133	Trihydroxycinnamoyl quinic acid	Nakamoto et al. (2020)
**3** ^**c**^	9.67	207	161	Ethylcaffeate	Nedamani et al. (2014)
**4** ^**c**^	13.17	353	191, 179, 173, 135	3‐*O*‐caffeoylquinic acid	Opara and Chohan (2014)
**5** ^**c**^	16.50	353	191, 179, 173, 135	5‐*O*‐caffeoylquinic acid	Opara and Chohan (2014)
**6** ^**c**^	18.07	367	193, 191, 173, 134	4‐feruloyl‐quinic acid	Pathan et al. (2011)
**7** ^**c**^	18.89	515	353, 191, 179, 173, 135	Di‐*O*‐caffeoylquinic acid	Opara and Chohan (2014)
**8** ^**c**^	20.18	499	377, 273, 163, 119	Caffeoyl‐coumaroyl‐quinic acid	Peana et al. (2006)
**9** ^**c**^	20.94	609	301	Quercetin‐3‐*O*‐rutinoside	Opara and Chohan (2014)
**10** ^**c**^	21.77	609	301	Quercetin‐*O*‐hexoside deoxyhexoside isomer 1	Opara and Chohan (2014)
**11** ^**c**^	22.10	609	301	Quercetin‐ hexoside deoxyhexoside isomer 1	Opara and Chohan (2014)
**12** ^**c**^	22.37	477	315, 300, 179, 151	Isoramnetin‐*O*‐hexoside	Pereira et al. (2007)
**13** ^**c**^	23.12	593	503, 473, 383, 353	6, 8‐C‐dihexosylapigenin 1	Piluzza and Bullitta (2011)
**14** ^**c**^	24.05	593	503, 473, 383, 353	6, 8‐C‐dihexosylapigenin 2	Piluzza and Bullitta (2011)
**15** ^**c**^	24.88	473	429,323, 161, 221	Apigenin‐acetyl‐hexoside 1	Pereira et al. (2007)
**16** ^**c**^	25.16	473	429,323, 161, 221	Apigenin‐acetyl‐hexoside 2	Pereira et al. (2007)
**17** ^**c**^	25.39	609	489, 369	Luteolin 6,8‐di‐C‐glucoside	Pereira et al. (2007)
**18** ^**c**^	37.03	329	285	Carnosol	Prachayasittikul et al. (2018)
**19** ^**c**^	38.25	431	311, 283	Apigenin‐C‐glucoside	Pereira et al. (2007)
**20** ^**c**^	39.61	925	605, 563, 443, 383, 353	Luteolin 7‐*O*‐(6″‐quinoyl)‐rhamnosyl‐6‐C‐pentosyl‐8‐C,O‐(6″acetyl)‐glucoside	Peana et al. (2006)

**TABLE 3:** Retention time (Rt), wavelengths of maximum absorption in visible region (λmax), mass spectral data, and tentative identification of phenolic compounds in *Allium sativum* L.

Peak	Rt (min)	[M + H]^+^ (m/z)	MS^2^ (m/z)	Tentative identification	References
1^al^	3.20	163	73	Allicin	Purkait et al. (2020)
2^al^	3.41	162	145,73	*S*‐allyl‐l‐cysteine	Purkait et al. (2020)
3^al^	6.61	178	88	Allin	Purkait et al. (2020)
4^al^	8.87	291	162,145,73	γ‐Glutamyl‐*S*‐allylcysteine	Purkait et al. (2020)
5^al^	12.20	234	89	Ajoene	Rakholiya et al. (2015)
6^al^	13.22	115	89	Diallyl sulfide	Renna et al. (2015)
7^al^	15.67	146	115,73,89	Diallyl disulfide	Renna et al. (2015)

**TABLE 4:** Retention time (Rt), wavelengths of maximum absorption in visible region (λ_max_), mass spectral data, and tentative identification of phenolic compounds in the extract mixture of *Allium sativum* and *Coriandrum sativum* L.

Peak	Rt (min)	[M − H]^−^ (m/z)	MS^2^ (m/z)	Tentative identification	References
**1** ^**c**^	0.93	377	341,215,179,332	Caffeoyl‐*O*‐hexoside	Mekni et al. (2013)
**2** ^**c**^	1.25	369	195, 191, 133	Trihydroxycinnamoyl quinic acid	Nakamoto et al. (2020)
**1** ^**al**^	3.20	161	71	Allicin	Purkait et al. (2020)
**2** ^**al**^	3.41	160	143,71	*S*‐allyl‐l‐cysteine	Purkait et al. (2020)
**3** ^**al**^	6.61	176	86	Allin	Purkait et al. (2020)
**4** ^**al**^	8.87	289	160,143,71	γ‐Glutamyl‐*S*‐allylcysteine	Purkait et al. (2020)
**5** ^**al**^	12.20	232	87	Diallyl sulfide	Rakholiya et al. (2015)
**6** ^**al**^	13.22	113	87	Allicin	Renna et al. (2015)
**4** ^**c**^	13.49	353	191, 179, 173, 135	3‐*O*‐caffeoylquinic acid	Opara and Chohan (2014)
**5** ^**c**^	15.28	353	191, 179, 173, 135	5‐*O*‐caffeoylquinic acid	Opara and Chohan (2014)
**6** ^**c**^	18.05	367	193	3‐feruloylquinic acid	Piluzza and Bullitta (2011)
**7** ^**c**^	18.82	515	353, 191, 179, 173, 135	Di‐*O*‐caffeoylquinic acid	Opara and Chohan (2014)
**8** ^**c**^	20.12	499	377, 273, 163, 119	Caffeoyl‐coumaroyl‐quinic acid	Peana et al. (2006)
**9** ^**c**^	20.83	609	301	Quercetin‐3‐*O*‐rutinoside	Opara and Chohan (2014)
**10** ^**c**^	21.65	609	301	Quercetin‐*O*‐hexoside deoxyhexoside isomer 1	Opara and Chohan (2014)
**11** ^**c**^	21.92	609	301	Quercetin‐hexoside deoxyhexoside isomer 1	Opara and Chohan (2014)
**12** ^**c**^	22.09	477	315, 300, 179, 151	Isoramnetin‐*O*‐hexoside	Pereira et al. (2007)
**14** ^**c**^	23.89	593	503, 473, 383, 353	6, 8‐C‐dihexosylapigenin	Piluzza and Bullitta (2011)
**15** ^**c**^	25.31	473	429,323, 161, 221	Apigenin‐*O*‐acetyl‐hexoside isomer 1	Pereira et al. (2007)
**16** ^**c**^	25.50	473	429,323, 161, 221	Apigenin‐*O*‐acetyl‐hexoside isomer 2	Pereira et al. (2007)
**18** ^**c**^	37.03	329	285	Carnosol	Prachayasittikul et al. (2018)
**19** ^**c**^	37.75	431	311, 283	Apigenin‐*C*‐glucoside	Pereira et al. (2007)
**20** ^**c**^	39.75	925	605, 563, 443, 383, 353	Luteolin 7‐*O*‐(6″‐quinoyl)‐rhamnosyl‐6‐C‐pentosyl‐8‐C,O‐(6″acetyl)‐glucoside	Peana et al. (2006)

Many of the tentatively identified compounds during the current study were previously reported by literature, such as in a cultivated (in vitro and in vivo) *C*. *sativum* vegetative parts and fruits characterized by HPLC‐DAD‐ESI/MS. Barros et al. (2012) reported that HPLC‐DAD‐ESI/MS phenolic acids such as the 5‐*O*‐caffeoylquinic acid and Di‐*O*‐caffeoylquinic acid and flavonoids and glycolyzed derivatives such as the quercetin‐3‐*O*‐rutinoside were found in Portuguese *C*. *sativum*. Besides, *A*. *sativum* hydroethanolic sample was mainly distinguished by the presence of organosulfur compounds and unstable thiol groups that presented a distinct fragmentation pattern when analyzed by HPLC‐Q‐TOF/MS–MS. Fragmentation pattern recorded in positive mode [M + H]^+^ allowed the identification of seven compounds as described in Figure 1 and Table 2. Peak 1al (Rt 3.20 min) was tentatively identified as allicin since a precursor molecular ion is obtained at m/z [M + H] + 163 and MS fragment signal was recorded at m/z 73. Peak 2al (Rt 3.4 min) presented pseudomolecular ion [M + H]^+^ at m/z 162 releasing two fragment ions at 145 and 73 to be assigned as *S*‐allyl‐l‐cysteine (Zhu et al., 2016). Peak 3al (Rt 6.61 min) was identified as allin basing on the fragmentation pattern [M + H]^+^ at m/z 178 and MS2 at m/z 88 by the loss of allyl methyl sulfide fragment. Peak 4al (Rt 8.87 min) was assigned as γ‐glutamyl‐*S*‐allylcysteine; this dipeptide showed a particular fragmentation pattern with a main deprotonated molecular ion at m/z 291 and MS fragments at m/z 162 (loss of allylcysteine), m/z 145 (loss of glutamyl group). Peaks 5al (Rt 12.20 min), 6al (Rt 13.22 min), and 7al (Rt 15.67 min) were tentatively identified as metabolites of the main previous compounds and Peak 5al is assigned to be ajoene being able to exhibit a deprotonated molecular ion at m/z 234 (Salem et al., 2017) and main fragment at m/z 89 corresponding to the loss of allyl methyl sulfide. Peaks 6al and 7al were tentatively identified as diallyl sulfide and diallyl disulfide (Nakamoto et al., 2020) respectively basing on the fragmentation pattern [M + H]^+^ at m/z 115/146 and MS2 main ion at m/z 89 [(M + H‐allyl sulfide]^+^). Nakamoto et al. (2020), Salem et al. (2017), and Zhu et al. (2016) reported similar compounds in some garlic samples which led to the conclusion that garlic is frequently rich in compounds in organosulfur compounds and certain amino‐acids (glutamate and cysteine) derivatives.

### 3.3 Phenolics and flavonoids content

Biochemical colorimetric assays were used to determine the amounts of TPC, TFC, and flavonols in the studied extracts, and results are presented in Figure 1. The tests allowed the estimation of 2.97 ± 0.09 and 8.63 ± 0.52 mg of GAeq/g extract, in GE and CE extracts, respectively. On the other hand, the mixture of garlic and coriander extract considerably improved the amounts of phenolic content by recording higher values (16.53 ± 0.28 mg of GAeq/g extract) due to a rise in the content of phenolic compounds (additive amounts). The same finding is reported in the results obtained for TFC and flavonols that recorded values ranging from 1.2 ± 0.13 to 2.01 ± 0.11 mg of extract Ceq/g extract and from 1.15 ± 0.08 to 4.29 ± 0.35 mg of extract Req/g extract respectively for garlic and coriander extracts. Here again, the combination extract was also enriched in TFC and flavonols and recorded highest values (4.18 ± 0.37 mg of extract Ceq/g extract and 7.13 ± 0.53 mg of extract Req/g extract, respectively). Previously, Shirazi et al. (2014) quantified the whole phenolics and flavonoids amount of three popularly used herbal and spice formulations (basil, cilantro, onion, ginger, and clove) and underlined that the highest level of polyphenols and flavonoids is always obtained with the formulations compared to each ingredient analyzed separately. For most of the cases, flavonoids represent a wide group of natural phenolic compounds that include also flavonols. Thus, according to the LC/MS analyses in the previous section, the main part of the phenolic profile of the CE and CGE extracts indicates the different classes of flavonoids/flavonols as validated here spectrometrically by the TFC/TF measures that were proportionally higher in those extracts.

### 3.4 Antioxidant activity

The antioxidant capacity of CE, GE, and CGE was assessed by measuring the EC_50_ values of the DPPH, FRAP, and β‐carotene bleaching assays, and results are mentioned in Table 5. According to the three antioxidant tests, CE exhibited the highest activities, in contrast to the GE extract. These findings correlated with the amount of total phenolic compounds which are known to be responsible for free radicals scavenging (Czemerys & Oszmian, 2007; Piluzza & Bullitta, 2011). Meanwhile, the combination of the two spices produced better EC_50_ values (0.09, 0.08, and 0.16 mg mL^−1^), therefore an improvement in the antioxidant activities. Accordingly, the antioxidant combination index (CI) calculated based on the EC_50_ values of garlic/coriander combination extract showed a synergistic effect (CI = 0.59, 0.46, and 0.76, respectively) with the three used antioxidant tests as depicted in Table 5. The same behavior of the combination effect was reported in several studies when combining common herbs and spices. Bag and Chattopadhyay (2015) have obtained synergistic effect (CI = 0.79 < 1) with coriander/cumin seed oil combination. Additionally, Mansour et al. (2016) have reported a synergistic antioxidant effect in clove/cinnamon combination (CI = 0.87 < 1). This synergistic mechanism was previously explained by Nedamani et al. (2014) as an electron‐transferring phenomenon from the compound of low antioxidant activity to the compound with higher antioxidant activity, and retrieval of this compound in order to donate a hydrogen group to another free radical and continue the process. Previous study showed that the currently characterized compounds such as the phenolic acids and flavonoid derivatives are potential antioxidants. The caffeoyl quinic acid (Gong et al., 2019), feruloyl‐quinic acid (Sarı et al., 2019), and caffeic acid derivatives (Tajner‐Czopek et al., 2020) were reported as antioxidant molecules through the position and the number of hydroxyl groups of the phenolic ring that are directly related to the free radical scavenging ability. Besides, flavonol glycoside compounds such as quercetin, apigenin, and luteolin‐*O*‐glycoside (highly present in the CE) are reported to be highly antioxidant compounds proportionally to the number of phenolic hydroxyl group through many scientific reports (Tian et al., 2021). Furthermore, organosulfur compounds in Allium vegetables including garlic and onion such as γ‐glutamyl peptides, allin, and allicin were reported (Kim et al., 2018) to have antioxidant capacities evaluated by oxygen radical absorbance capacity (ORAC), DPPH, and 2,2′‐azinobis (3‐ethylbenzothiazoline‐6‐sulfonic acid) (ABTS) radical scavenging assays.

**TABLE 5:** Phytochemicals and antioxidant activities of garlic, coriander and their combination extracts and antioxidant combination index (CI).

	Antioxidant activity EC_50_ (mg mL^−1^)			Combination indexes			Type of interaction
	*Coriandrum sativum*	*Allium sativum*	Combination	CI_1_	CI_2_	CI	
DPPH	0.22 ± 0.04	0.47 ± 0.05	0.09 ± 0.002	0.19	0.40	0.59	Synergistic
FRAP	0.26 ± 0.05	0.48 ± 0.05	0.08 ± 0.003	0.16	0.30	0.46	Synergistic
β‐Carotene Bleaching	0.32 ± 0.03	0.61 ± 0.07	0.16 ± 0.01	0.26	0.5	0.76	Synergistic

### 3.5 Antimicrobial activity

Currently, there is a growing concern to target natural antimicrobial compounds being safe to use as preservatives (Katarzyna & Katarzyna, 2016; Ziani et al., 2020). The antimicrobial activity of the studied spices was explored on common foodborne microorganisms, including bacteria and yeasts, and results of MICs, MBCs, MFCs, and FICI are depicted in Table 6. Globally, the assessed extracts exhibited a significant in vitro growth inhibition against the studied microorganisms. The CE showed MIC values varying from 0.2 to 0.8 mg mL^−1^ and from 0.8 to 3.12 mg mL^−1^ when the GE exhibited MIC values varying from 0.2 to 1.6 mg mL^−1^ and from 0.2 to 6.25 mg mL^−1^ against bacterial and yeast strains, respectively. While, for the combination extract, average MIC values varied from 0.4 to 0.8 mg mL^−1^ and from 0.2 to 3.12 mg mL^−1^ for bacterial and yeast strains, respectively, suggesting a synergism action that appeared to increase antimicrobial capacity compared to the individual extracts. However, the studied extracts have also been found to show bactericidal/fungicide activity versus the tested strains through the recorded MBC and MFC values (Table 6).

**TABLE 6:** Antibacterial, antifungal activities (MIC, MBC and MFC in mg mL^−1^) and fractional Inhibitory Concentration Index (FICI) of garlic, coriander and their combination extracts.

Bacterial strains	*Coriandrum sativum*		*Allium sativum*		Combination					Ampicillin	
	MIC	MBC	MIC	MBC	MIC	MBC	FIC	FICI	Interaction	MIC	MBC
*Listeria monocytogenes* CECT933	0.2	25	0.4	0.8	0.4	0.8	1 (A) 2 (B)	3	Additive	0.08	3.0
*Staphylococcus aureus* ATCC25923	0.2	12.5	0.4	0.8	0.8	3.12	2(A) 4 (B)	6	Antagonistic	0.08	0.625
*Bacillus cereus* ATCC 11778	0.4	6.25	0.2	0.4	0.4	0.8	2 (A) 1 (B)	3	Additive	0.08	0.625
*Enterococcus epidermidis* CECT231	0.2	0.4	0.8	1.6	0.4	0.8	0.5 (A) 2 (B)	2.5	Additive	0.2	0.625
*Salmonella enterica subsp*. *Enterica* ECT443	0.4	1.6	0.2	0.4	0.8	3.12	4 (A) 2 (B)	6	Antagonistic	0.2	3.0
*Escherichia coli* ATCC35218	0.2	50	0.4	0.8	0.4	0.8	1 (A) 2 (B)	3	Additive	0.02	3.0
*Shigella flexneri* CECT 4804	0.2	0.8	0.8	3.12	0.4	1.6	0.5 (A) 2 (B)	2.5	Additive	0.2	6.0
*Pseudomonas aeruginosa* PAO1	0.8	12.5	1.6	12.5	0.8	1.6	0.5 (A) 1 (B)	1.5	Additive	0.1	12.0

Yeast strains	*Coriandrum sativum*		*Allium sativum*		Combination					Amphotericin B	
	MIC	MFC	MIC	MFC	MIC	MFC	FIC	FICI		MIC	MBC
*Candida parapsilosis* ATCC 20019	1.6	25	6.25	12.5	3.12	50	0.49 (A) 1.95 (B)	2.44	Additive	0.2	0.39
*Candida albicans* ATCC 2019	3.12	1.6	0.4	0.4	0.8	50	2 (A) 0.25 (B)	1.12	Additive	0.026	0.82
*Candida krusei* ATCC 6258	1.6	6.25	0.4	12.5	0.8	12.5	1 (A) 0.25(B)	1.25	Additive	0.1	0.2
*Candida tropicalis* 06–85	0.8	3.12	0.2	0.2	0.2	0.2	1 (A) 0.25 (B)	2.25	Additive	0.42	6.75

*Listeria monocytogenes*, *S*. *aureus*, *E*. *epidermidis E*. *coli*, and *S*. *flexneri* were the most sensitive germs and *P*. *aeruginosa* was the most resistant bacteria. Whereas the yeast strains were less sensitive toward CE and more sensitive to the GE with average values for the CGE suggesting an additive effect supported probably by the garlic extract (positive interaction). Accordingly, the recorded in vitro FICI indexes allowed to classify the combination of garlic plus coriander as additive (1.5 < FICI < 4) against all used strains except *S*. *aureus* and *S*. *enterica* where the combination was classified as antagonistic (FICI > 4). Meanwhile, this additive effect was more pronounced against *P*. *aeruginosa* (FICI = 1.5) and *C*. *albicans* (FICI = 1.12). The obtained values may be correlated to a synergic interaction of the chemical compounds determined in the extracts. A similar study was conducted by Baljeet et al. (2015) who reported an additive effect and no synergistic one of the combined extracts of cumin (*Cuminum cyminum*), ginger (*Zingiber officinale*), and garlic (*A*. *sativum*) against *B*. *subtilis*, *P*. *fluorescens*, *S*. *typhi*, *C*. *albicans*, and *R*. *azygosporus*. Additionally, similar results were obtained with combination of coriander and cumin essential oils at low concentrations (Bag & Chattopadhyay, 2015). In fact, these authors reported that the combinations have additive activity on *M*. *luteus* and *S*. *typhimurium*. While the same combination possessed synergistic activity against the following foodborne bacteria: *B*. *cereus*, *L*. *monocytogenes*, *S*. *aureus*, and *E*. *coli* (Bag & Chattopadhyay, 2015). Additive interaction was also reported against the same bacteria when using the combination of coriander/mustard, and cumin/mustard essential oils. Previously, Pereira et al. (2007), Sarı et al. (2019), and Stojković et al. (2013) reported feruloylquinic acid, caffeoylquinic acid, and caffeic acid derivatives and their isomers as antimicrobial molecules suggesting that the currently highlighted antimicrobial activity to be a structure‐dependent activity since an additive effect was reported with the CGE. The response to organosulfur compounds of garlic, with the sulfur (thiol) groups of *Candida* species (sensitive to GE), could be done by interaction with microbial proteases, causing growth inhibition (Wilson & Demmig‐Adams, 2007). The profiled compounds of the CGE could induce microbial membrane damage by a synergistic mechanism and cause the cell membrane integrity loss and therefore the cell morphology alteration by binding to cell wall polypeptides, surface‐exposed adhesions, and membrane‐bound enzymes.

### 3.6 Acute toxicity study

Throughout the duration of the study (14 days), the tested doses (100, 200, and 500 mg kg^−1^) of all the extracts showed no deleterious effects and no change in general behavior (e.g., salivation, tremors, and diarrhea). Likewise, no mortality of the test subjects was recorded. According to Chung (2006), when the LD_50_ was less than 10 mg kg^−1^ or greater than 50 mg kg^−1^ of body weight, plant extracts were respectively considered highly toxic or non‐toxic. Thus, the LD_50_ values of EC, GE, and CGE would be well above 500 mg kg^−1^ and could be suggested as safe for ingestion. Although an LD50 above 500 mg kg^−1^ indicates relatively no acute toxicity, this does not necessarily mean these extracts can be safely consumed long term. In fact, evaluating acute toxicity alone is not sufficient to fully characterize the safety profile of these botanical extracts.

To better understand their potential for safe use, it is essential to conduct sub‐chronic (90‐day) and chronic (6‐month) toxicity studies in accordance with well‐established guidelines, such as those recommended by the OECD. These studies would allow assessing the potential for adverse effects with repeated dosing over extended periods, determining a no observed adverse effect level (NOAEL), and evaluating impacts on a broad range of biochemical, hematological, and histopathological parameters. Only by completing these more comprehensive toxicological evaluations can we reliably conclude whether these extracts can be recommended for safe long‐term consumption. The authors are currently planning these sub‐chronic and chronic toxicity studies to fill these data gaps and thoroughly assess the safety profile of these extracts.

### 3.7 Analgesic activity

The analgesic effect of the extracts was assessed by induction of the contortion reflex in rats following the experimental procedure described by Firoj et al. (2006). The ache induced by an acetic acid injected in the peritoneal cavity of the mice arises from an inflammatory response. The reflexes of the posterior parts and movement of the legs were calculated to perform a quick evaluation of peripheral analgesic effect, and the results of writhing are presented in Table 7. The pain sensation was induced by a localized inflammatory response due to the release of several mediators such as free arachidonic acid from tissue phospholipids via cyclooxygenase (COX), and prostaglandin biosynthesis into the peritoneum. These mediators were involved in the stimulation of nociceptive neurons (Firoj et al., 2006). When compared to the untreated group, the obtained results showed that with all the assessed doses of extracts, the treated groups exhibited a remarkable decrease (*p* < .05) in the writhes numbers. Thus, an analgesic activity of CE, GE, and CGE was noted (statistically significant, *p* < .05) in a dose‐dependent manner. Interestingly, when subjects received a 200 mg kg^−1^ dose of CE and GE, a very marked reduction in writhing was observed with 79.94% and 84.86% inhibition, respectively. These values were of the same order of the positive control group treated with ASL (80.92% of writhing reduction). The combination as for it, allows to record better values (91.85%) higher even than those of ASL, this indicates a marked synergetic effect. In the literature, Pathan et al. (2011) have already pointed out a significant analgesic activity of the aqueous extract of *C*. *sativum* at 50, 100, and 200 mg kg^−1^, using the hot plate method, and extracts demonstrated analgesic activity in comparison with to control group. Furthermore, the abundance of phenolic acids such the caffeoylquinic acid, feruloyl‐quinic acid, and caffeic acid derivatives and flavonols glycoside compounds (quercetin, apigenin, and luteolin‐*O*‐glycosides) as well as monoterpenic compounds (linalool) in *C*. *sativum* alongside the organosulfur compounds in *A*. *sativum* and their co‐presence in the combination may assume a synergistic effect of all these molecules to inhibit the inflammatory process responsible for the induced analgesic effect. Thus, according to previous reports, the mechanism of action of those molecules was linked to COX inhibition and to the decrease of iNOS enzyme activity and thereafter the NO release (Guo et al., 2014; Sarı et al., 2019). Moreover, Peana et al. (2006) attributed the analgesic potential to the inhibition of NO synthesis and COX activity by the aspirin‐like effect. Similarly, it was reported that some major phytoconstituents of garlic such as allicin, diallyl disulfide, diallyltrisulfide, and ajoene (Shang et al., 2019) may inhibit cyclooxygenase peripherally and act on opioid receptors centrally leading to analgesia. Therefore, despite the lack of data on the analgesic activity of spices combination extracts, certain authors have reported the positive effect of the combination of herbs or spices with different analgesic drugs (Wu & Wu, 2014).

**TABLE 7:** Analgesic and gastro‐protective activities of garlic, coriander and their combination extracts.

	Analgesic activity			Gastro‐protective	
Extracts	Concentration (mg Kg^−1^)	Number of writhes	Inhibition of writhing (%)	Ulcer index (mm)	Inhibition (%)
*Coriandrum sativum*	50	40.66 ± 0.59**	49.03	94.20 ± 4.02***	46.11
	100	32.00 ± 1.57**	59.89	61.30 ± 3.74***	64.93
	200	16.00 ± 1.09***	79.94	38.04 ± 4.12***	78.24
*Allium sativum*	50	37.00 ± 0.36**	53.63	72.13 ± 2.83***	85.74
	100	24.66 ± 1.33**	60.08	45.80 ± 3.10***	73.80
	200	12.08 ± 0.51***	84.86	25.60 ± 2.88***	85.35
Combination	50	31.16 ± 0.32**	60.94	20.40 ± 3.44***	88.33
	100	20.10 ± 0.21**	74.93	10.80 ± 3.50***	93.82
	200	6.50 ± 0.63***	91.85	5.60 ± 2.85***	96.79
Control	–	79.80 ± 0.62	–	174.82 ± 4.22***	–
ASL	200	15.22 ± 0.42***	80.92	–	–
Omeprazole	30	–	–	11.64 ± 0.52***	93.34

*Note*: Values are expressed as mean ± S.E.M. (*n* = 6).

Abbreviation: ASL, acetylsalicylic acid.

** *p* ≤ .05 significant versus negative control by post hoc Dunnett's test.

*** *p* ≤ .05 significant versus positive control by post hoc Dunnett's test.

### 3.8 Gastroprotective activity

**FIGURE 2:**
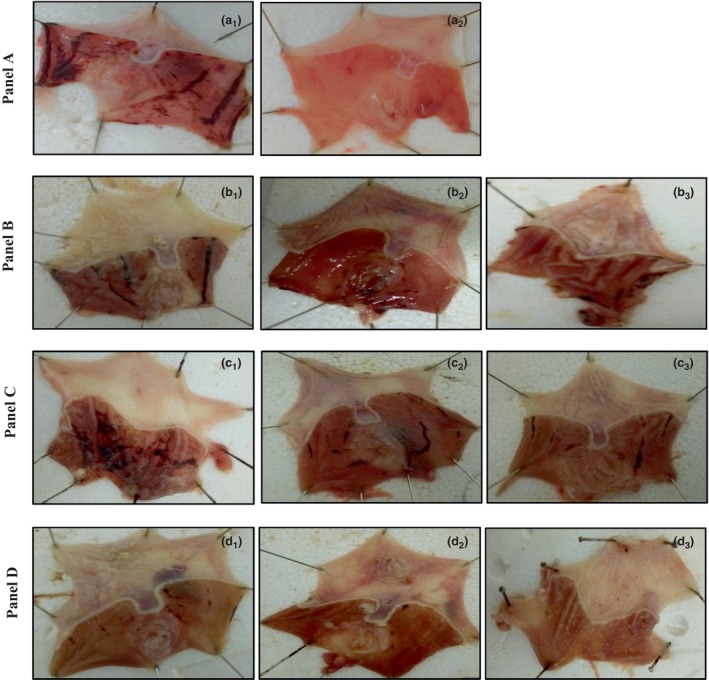
Macroscopic appearance of the gastric lesions in rats. (a): (a_1_) the stomach of control rats, (a_2_) the stomach of rats treated with omeprazole (30 mg kg^−1^). (b) (b_1_–b_3_) the stomachs of rats treated with *Allium sativum* (50, 100, and 200 mg kg^−1^, respectively). (c) (c_1_–c_3_) the stomachs of rats treated with *Coriandrum sativum* (50, 100, and 200 mg kg^−1^, respectively). (d) (d_1_–d_3_) the stomachs of rats treated with Combination (50, 100, and 200 mg kg^−1^, respectively).

The gastric ulcer induced by EtOH/HCl is one of the models commonly used to study the possible protection of the gastric mucosa by herbal extracts. This mixture of chemicals causes harmful damage to the gastric mucosa by inducing necrosis and tissue damage (Baiubon et al., 2016). This test was adopted during the current research to highlight the gastroprotective potential of the extracts analyzed, and the measurements of the ulcer index are reported in Table 7. Mitigation of lesions when extracts are used as well as omeprazole gastroprotection are shown in Figure 2. Preventive intraperitoneal administration of CE and GE (50, 100 and 200 mg kg^−1^) exhibited a dose‐dependent prophylactic activity on HCl/EtOH‐induced gastric mucosa lesion with 38.04 mm (78.24%) to 25.60 mm (85.35%) of ulcer index, respectively, when 200 mg kg^−1^ is administrated significantly, the pre‐treatment with CGE, at all tested‐dose, (*p* < .01) provide more effective gastroprotective activity where, 100 and 200 mg kg^−1^ of CGE gives an inhibition percentage of 93.82% and 96.79%, respectively, which were comparable to the omeprazole (93.34%) at a dose of 30 mg kg^−1^. Accordingly, a synergistic effect is here again noted between the two spices to induce a gastroprotective action. As presented in Figure 2, the HCl/EtOH gavage to the rats provoked serious mucosal damage associated with various extents of hemorrhagic bands. Thus, when extracts were administrated, the lesions were remarkably low in appearance compared to the control groups who did not receive the extracts. According to Al‐Mofleh et al. (2006), the mechanism of herb‐induced gastro protection may be related to their cytoprotective, antisecretory, and antioxidant activities. In previous studies, it was demonstrated that coriander alone is an inherent herb to preserve cells mucosa from gastric injury by scavenging the reactive oxygen species or probably by forming a protective layer by hydrophobic interactions (Al‐Mofleh et al., 2006). The prophylactic effect of garlic in rat‐induced ulcer was also reported (Tope, 2014). Garlic organosulfur compounds such as S‐allylcysteine, S‐allylmercaptocysteine, and diallylsulfide may act together to stimulate an increase in mucous cell count thereby increasing mucus secretion. It is worth to note that there was a synergistic interaction between the bioactive components in both spices approved by the in vivo test in this study. This could explain the observed antiulcer activity of CGE.

## 4 CONCLUSIONS

The outcome of the current research suggested that the combination of CE and GE indicated as CGE can exhibit some synergistic effects observed when tested in vitro (antioxidant and antimicrobial activities) and in vivo (analgesic and gastroprotective activities). CGE was more effective in the proposed bioassay test showing a correlation of the observed activities with the content of phenolic compounds. Chromatographic analysis profiled caffeoylquinic acid, feruloyl‐quinic acid, caffeic acid derivatives and flavonol glycoside compounds (quercetin, apigenin, and luteolin‐*O*‐glycosides) as well as monoterpenic compounds (linalool, α‐pinene, and γ‐terpinene) in *C*. *sativum*, and a long side the organosulfur compounds (allicin, *S*‐allyl‐l‐cysteine, allin, γ‐Glutamyl‐*S*‐allylcysteine and allyl sulfide) in *A*. *sativum*. The profiled compounds were co‐present in CGE. The combination also induced significant analgesic and anti‐inflammatory activities in vivo with significant dose‐dependent effects close to chemical standards especially at the dose of 200 mg kg^−1^. The CI and FICI indices overall suggested an additive effect in the garlic/coriander combination. Therefore, this work could be considered as a line of research to be complemented by more in‐depth pharmacological studies.

## AUTHOR CONTRIBUTIONS

Conceptualization: M.A.B., H.C., M.B.M., B.S, and H.M.; methodology: M.A.B., H.C., B.E.C.Z., M.B.M., K.B., G.F., and H.M.; software: M.A.B., H.C., A.B.H., and H.M.; validation: M.A.B., H.C., M.S., M.B.M., B.S., M.K., and H.M.; formal analysis: B.E.C.Z., M.S., and K.B.; investigation: M.A.B., H.C., B.E.C.Z., M.S., W.M., K.B., B.S., A.B.H., and H.M.; resources: W.M., M.B.M., G.F., and H.M.; data curation: M.A.B., H.C., B.E.C.Z., M.S., M.B.M., K.B., and B.S.; writing – original draft preparation: M.A.B., H.C., and H.M.; writing – review and editing: W.M., M.B.M., K.B., B.S., A.B.H., M.K., and G.F.; visualization: M.S., M.B.M., K.B., M.K., and B.S.; supervision: W.M., MZ., G.F., and H.M.; project administration: W.M., and H.M.; funding acquisition: W.M. and H.M. All authors have read and agreed to the published version of the manuscript.

## CONFLICT OF INTEREST STATEMENT

The authors declare no conflict of interest.

## Data Availability

The results presented in this study are accessible on request from the first author.
